# A Review of Techniques for RSS-Based Radiometric Partial Discharge Localization

**DOI:** 10.3390/s21030909

**Published:** 2021-01-29

**Authors:** David W. Upton, Keyur K. Mistry, Peter J. Mather, Zaharias D. Zaharis, Robert C. Atkinson, Christos Tachtatzis, Pavlos I. Lazaridis

**Affiliations:** 1 Swedish Institute of Space Physics, SE-981 Kiruna, Sweden; david.upton@irf.se; 2 Oxford Space Systems, Harwell OX11 0RL, UK; keyur.mistry@oxford.space; 3 Department of Engineering and Technology, University of Huddersfield, Huddersfield HD1 3DH, UK; p.j.mather@hud.ac.uk; 4 Department of Electrical and Computer Engineering, Aristotle University of Thessaloniki, 54124 Thessaloniki, Greece; zaharis@auth.gr; 5 Department of Electronics and Electrical Engineering, University of Strathclyde, Glasgow G1 1XW, UK; robert.atkinson@strath.ac.uk (R.C.A.); christos.tachtatzis@strath.ac.uk (C.T.)

**Keywords:** field trials, localization algorithm, partial discharge, radiometric detection, RSS, WSN

## Abstract

The lifespan assessment and maintenance planning of high-voltage power systems requires condition monitoring of all the operational equipment in a specific area. Electrical insulation of electrical apparatuses is prone to failure due to high electrical stresses, and thus it is a critical aspect that needs to be monitored. The ageing process of the electrical insulation in high voltage equipment may accelerate due to the occurrence of partial discharge (PD) that may in turn lead to catastrophic failures if the related defects are left untreated at an initial stage. Therefore, there is a requirement to monitor the PD levels so that an unexpected breakdown of high-voltage equipment is avoided. There are several ways of detecting PD, such as acoustic detection, optical detection, chemical detection, and radiometric detection. This paper focuses on reviewing techniques based on radiometric detection of PD, and more specifically, using received signal strength (RSS) for the localization of faults. This paper explores the advantages and disadvantages of radiometric techniques and presents an overview of a radiometric PD detection technique that uses a transistor reset integrator (TRI)-based wireless sensor network (WSN).

## 1. Introduction

A widely used and very effective metric for assessing the condition of high voltage (HV) plants is partial discharge (PD). PD is an electrical fault that occurs within the insulating material of high voltage plant equipment, such as transformers, switch gear and transmission lines [1,2]. PD is caused due to several reasons, such as defects in manufacturing, defects during installation, ageing and deterioration, over-stressing in service, corona discharge, surface discharge, and cavities. One frequent causes of PD is by a decrease in permittivity, and therefore an increase in electric field strength, due to the presence of a void or defect within the dielectric, resulting in a discharge across the void that does not breach the conductors [3,4], but can worsen over time due to prolonged electrical stresses increasing the physical size of the void. PD is defined by the IEC60270 standard as: “a localized electrical discharge that only partially bridges the insulation between conductors and which can or cannot occur adjacent to a conductor. Partial discharges are in general a consequence of local electrical stress concentrations in the insulation or on the surface of the insulation. Generally, such discharges appear as pulses having a duration of much less than 1 microsecond” [5].

These discharges may increase over time if the defect becomes physically larger, due to damage such as treeing (cracking of the insulation surrounding the void) caused by prolonged arcing across the void, eventually leading to catastrophic failure when the void is so large that the potential is able to breach the inner and outer conductors (referred to as flashover) [6]. PD faults are more likely to occur in aged HV plants, where long-term exposure to constant changes in environmental conditions may accelerate deterioration of the insulating material, such as thermal expansion and retraction.

**Figure 1:**
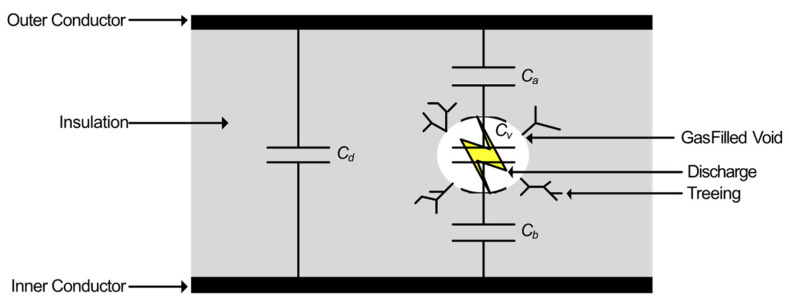
Gemant and Phillippoff gas-filled void partial discharge (PD) model [7].

An example of a typical PD fault is a gas-filled void within the insulation of a HV transmission line, represented by the Gemant and Phillippoff model [7] shown in Figure 1.

**Figure 2:**
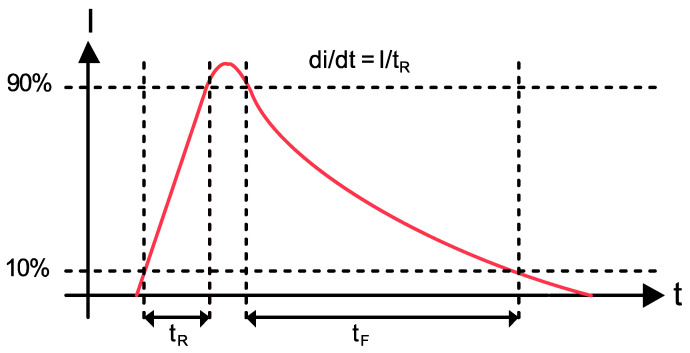
A PD current pulse across an HV insulation void.

Cd is the capacitance of the insulation, Cv is the capacitance of the gas-filled void, and Ca and Cb, are the dielectric capacitances in series with the void. As stated earlier, the permittivity of the void is lower than that of the surrounding insulation since the void tends towards the permittivity of air which is lower than that of the insulation; therefore, the electric-field strength within the void is increased and arcing may occur as the AC potential is increased across the conductors. The resulting arcing can weaken the surrounding insulation causing treeing, eventually leading to an increase in the size of the void and a greater risk of catastrophic failure. Figure 2 shows an example of a PD current pulse discharged across an insulation void as a result of the increased field strength within the void [8], where the strength of the PD is quantified by the total charge within it. Typically, the current pulse lasts between 1 and 1000 ns [8,9].

Detecting and monitoring PD can give an indication of the current condition of the fault and allows for any changes in the condition to be critically assessed over time. Indication that a fault is worsening allows for action to be taken before total failure occurs. Traditional techniques for detecting partial discharge, which include galvanic contact devices, high-frequency current transformers (HFCTs) and transient earth voltage (TEV) sensors [10,11], provide highly accurate and detailed information on any PD activity within the equipment under observation, due to the close coupling of the sensor to the piece of the plant, such as a transformer or switchgear. This information includes apparent charge and spectra, giving an indication of the type of PD fault, as well as any fault progression over time.

Due to the requirement of close coupling to each piece of the plant to be observed, however, each sensor is only capable of monitoring the equipment it is coupled to, requiring separate sensors for each item of the plant to be monitored. Therefore, extensive monitoring of HV equipment within a large-scale substation would be difficult using these techniques, due to the extensive wiring harnessing required, along with the complexity of the data acquisition system needed. In addition, reconfiguration of the system, if required, would be difficult, and likely costly, due to the amount of manual labor required to physically reconnect sensors, along with the design and cost of a replacement wiring harness. However, wired techniques are used in several industries because direct coupling to devices is preferred in several applications where monitoring of a selective single component is required. Futhermore, in many applications, direct coupling is preferred just because of being more sensitive and noise/interference immune. Even this way, it may still be complicated to have sufficient sensitivity to detect and locate the fault within a component. For example, in the case of transformers with metallic enclosures, having to recognize and locate PD sources within the transformer requires that the sensors be placed inside the enclosure, as hardly any signal can propagate through the bushings.

Various alternative techniques have been suggested and developed for PD detection, monitoring, and measurement; these include optical measurement [12], acoustic techniques [13], and radiometric techniques that utilize the far-field electromagnetic wave propagated from the PD source due to the current discharged over the void. Optical PD measurement involves using fiber optic sensors and probes to detect ionization in oil and gas dielectrics [14] by detecting the light emitted during the ionization process. The benefits of optical PD measurement techniques include immunity to electromagnetic interference, as well as isolation from the HV equipment, due to the use of light coupling. However, a disadvantage is the requirement that each sensor has to be physically connected to the plant under test.

Acoustic techniques involve capturing localized acoustic emissions from the source of PD via audio sensors [15]. Captured acoustic emissions can be cross correlated with known PD transient acoustic patterns in order to assess if the acquired signal is of PD origin [16]. As with optical detection, acoustic techniques have the advantage of immunity to electromagnetic radiation. A difficulty with this type of measurement is susceptibility to local ambient noise. Additionally, acoustic measurements suffer due to the unpredictability of propagation paths and attenuation, limiting the maximum dielectric thickness that can be measured [17].

**Figure 3:**
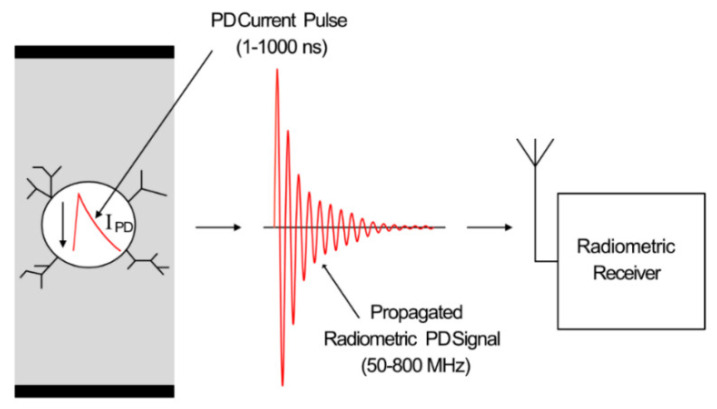
Radiometric PD propagation and reception using a radio receiver.

Radiometric PD measurement utilizes the electromagnetic signal that is propagated from a source of PD due to the current across the void. This radiometric signal is measured using a radio-receiver placed at a certain distance from the PD source. Figure 3 shows the process of receiving a radiometric signal from a source of PD.

The 1–1000 ns current pulse discharged across the void transmits an electromagnetic wave, which has a frequency range of approximately 50–3000 MHz, depending on the type of defect and structure of the void [18]. However, due to the low-pass effect of the band-limiting propagation environment [19], the frequency range is limited to a range of approximately 50–800 MHz [20]. This radiometric signal is then received and measured via a radio receiver. Radiometric PD detection has various advantages, such as ease of installation, comparatively low cost, and potential scale-ability. Various disadvantages include susceptibility to electromagnetic interference, including locally transmitted radio signals, and limited range due to sensor sensitivity and the propagation environment.

It should be noted that the PD diagnostic techniques explained above are still widely used in several industries; however, the selection of a particular technique depends on the diagnostic requirement and applications. This paper concentrates on radiometric PD detection techniques only, where an approximate location of PD is detected in a broad area.

## 2. Techniques for Radiometric Partial Discharge Measurement

Radiometric PD measurement has seen many developments over the past 20 years, due to the ease of installation and reconfiguration over other PD measurement techniques. Radiometric PD detection utilizes broadband radio receivers to detect and measure the electromagnetically radiated UHF signal propagated from a PD source [21,22,23]. Generally, multiple radiometric sensors, separated by a spacing of at least several meters, can be used to detect and locate a source of PD [24]. Radiometric PD detection allows for easy, non-intrusive installation, and is simple to reconfigure if required [22]. It provides an alternative that has several advantages over traditional PD measurement techniques since it does not require galvanic or physical contact with the item under observation; therefore, a single radiometric sensor is capable of monitoring multiple items of HV equipment simultaneously. Some wireless approaches for locating and monitoring PD utilize the received signal strength (RSS) of the electromagnetic energy propagated from a PD source, whilst more advanced techniques locate PD using the received time differences for a set of measurement sensors, such as time of arrival (TOA) and time difference of arrival (TDOA). Each technique has advantages and limitations, in relation to cost, complexity, accuracy, and efficiency.

### 2.1. Characteristics of Radiometric PD

As previously stated, the charge displaced in an insulation fault results in an electromagnetic signal propagated away from the discharge source. Whilst the actual current discharged in the fault is a pulse with a fall time in the order of 1 to 1000 ns, the resulting radiometric signal bears similarities to a classical decaying oscillation [25], with a frequency range in the order of 50–3000 MHz [26]. The frequency of the radiometric signal is dependent on the resonant structure of the insulation defect, resulting in a narrow-band band-pass response [27]. The propagation environment effectively low-pass filters this frequency range to 50–800 MHz, with the majority of the frequency content residing below 300 MHz.

**Figure 4:**
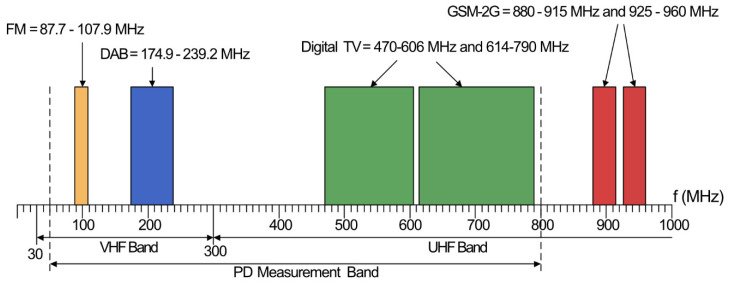
VHF/UHF electromagnetic spectrum with typical wide range transmission signals.

A variety of radio receivers are capable of detecting and measuring the transmitted PD signal; however, a difficulty of radiometric PD monitoring is susceptibility to any electromagnetic interference. This includes locally transmitted signals within the VHF and UHF bands, such as FM, private mobile radio, digital audio broadcasting (DAB) and digital TV. Figure 4 shows the typical spectrum of broadcast frequencies within the VHF and UHF bands.

FM occupies the 87.7–107.9 MHz band, whilst DAB is typically broadcast within 174–239.2 MHz. Digital TV is broadcast at from 470 to 606 MHz and 614 to 790 MHz. 2G GSM is broadcast at 880–915 MHz and 925–960 MHz; however, this is outside of the band of interest. There are also sources of interference transmitted locally, such as fluorescent lamp ignition [28], unlicensed portable radios, such as PMR446, and licensed portable and amateur radio. Fluorescent lamp ignitions should only be a possible issue in indoor environments, in which additional filtering may have to be employed to remove the interference if it is problematic.

Amateur radio, which is transmitted within bands of 50–52 MHz, 70–70.5 MHz, 144–146 MHz, and 430–440 MHz [29], requires a license for broadcasting. Radios are also restricted to a maximum transmission power of 10, 17, and 26 dBW for foundation, intermediate and full license types [30], corresponding to theoretical distances of 10, 22, and 63 km, respectively, for a receiver sensitivity down to −40 dBm. However, in reality, these distances would be reduced due to the complexity of the propagation path, and it is unlikely that most radio amateurs would transmit at the maximum power allowed. The radiation pattern of the antenna used will also decrease the likelihood of amateur transmission, causing interference to radiometric PD detection.

License-free PMR446 radio is broadcast in a narrow-band of 446.0–446.2 MHz, and has a maximum transmission power limit of −3 dBW [31], corresponding to a distance of approximately 2 km. Whilst it is unlikely that these transmissions outside of a radiometric PD monitoring area would be received, there is a possibility that personnel within, or visiting, a HV site may operate personal radios operating in this band. Such transmissions would not be a significant issue, since these types of transmissions would only be temporary, and could be easily discriminated from a source of PD. Interference of this type from outside of the monitoring area would also be easily discriminated when localization was applied, positioning it outside of the HV area.

Ensuring that these locally transmitted signals do not interfere with the radiometric PD measurement requires some knowledge of the typical frequency content of various types of radiometric PD signals, such as those that propagate from insulation faults within transformers, switchgear, and transmission lines. Measured spectra for a variety of simulated defects have been presented in publications for purposes including propagation effects on UHF PD, antenna comparisons for reception, and similarities between galvanic and radiometric PD signals.

The data presented in [32] measure the frequency response of radiometric PD within gas-insulated switchgear (GIS) using sulphur-hexaflouride (SF6). The measurements were made using a biconical antenna and a log-periodic antenna, with bandwidths of 30–300 MHz and 80–1000 MHz respectively, at a distance of 2 m from the source of PD. The reported spectra were between 30 and 820 MHz, with the majority of the energy from 30 to 75 MHz. In [33], radiometric PD spectra were measured for an air-blast circuit breaker, with a focus on the change in spectra due to the propagation environment. Three sensor positions were used, two of which were fixed at a distance of 12 m from the PD source, and one which was varied between 12 and 18 m. The mean spectra measured at each position fluctuated by over 50 MHz between the three sensor positions; however, the majority of the frequency content was still between 50 and 400 MHz at each position.

In [34] and [35], a PD fault was emulated using a sandwich of transformer paper submerged in transformer oil. Five sheets were used in [34] in between two electrodes paper, whilst eleven sheets were used in [35], with the five inner most sheets pierced with a 1 mm hole to produce an air-filled void. The radiometric PD signal produced was then measured using four different antennas, a 5 and 10 cm monopole, a log-periodic and a zig-zag antenna. The cumulative power was measured with and without the PD source in bands of 0–300 MHz and 1300 to 1900 MHz, with the ratio of the two cumulative quantities provided, therefore allowing for the difference in received power to be determined between background broadcast interference and PD within these bands, along with the power level in each band.

The results reported in [34] show that the majority of the received power is within the 0–300 MHz band, with the exception of the 5 cm monopole antenna, where the majority of received power was in the 1300–1900 MHz band. However, the reason for this is that the lower bandwidth of such an antenna is restricted due to its physical size, since a monopole antenna should be half the wavelength of the received signal frequency [36].

The results shown in [35] divide the measured spectrum into 250 MHz bands for each antenna, from DC to 2.5 GHz, with the log-periodic taken as the absolute measurement due to its flat frequency response across the specified range. The antennas were placed at a distance of 45 cm from the PD source, with the exception of the log-periodic antenna, which was placed at a distance of 90 cm to ensure the antenna was in the far field. Approximately 75% of the received power was measured in the 0–250 MHz band, along with 17% in the 250–500 MHz band. The remaining energy was between 1.4% and 0.26% in each consecutive frequency band.

The spectra of three simulated PD faults within a HV transformer were measured in [37]—an internal void PD fault, a surface PD fault and a insulator bushing PD fault. The internal PD fault was simulated with a dielectric oil-filled glass vessel placed inside a transformer tank which contained eleven layers of insulation, with a 1 mm hole perforated through the three inner most layers to produce an air-filled void. Two electrodes are connected to each side of the vessel to attach a HV source, the air was removed using a vacuum in order to ensure a lower permittivity withing the void than in the insulating layers.

The surface PD fault was created using a twisted-pair of HV, resin polyamide-imide insulation, enameled wire placed within the tank, which has an additional layer of polyester tris hydroxyethyl isocyanurate over the insulation. Finally, the bushing fault was generated using a porcelain bushing insulator whose surface was covered with a saline solution, which was allowed to dry in order to obtain a salt polluted surface to generate PD. The emulated PD faults were measured using two 10 cm monopole antennas, one placed inside the tank and one outside at a distance of 30 cm from the fault under test. The spectra were measured within three bands—a lower band of 0–300 MHz, a middle band of 300–1200 MHz, and an upper band of 1200–2500 MHz.

The cumulative power within each band was measured, with and without the PD fault, to determine the contribution of locally transmitted interfering transmissions. For the internal PD fault, the percentages of power measured by the inner antenna were 0.3%, 94.3%, and 5.4% for the lower, middle, and upper bands, respectively, whilst the percentages were 61.9%, 35.8%, and 2.1% in the lower, middle, and upper bands for the outer antenna, respectively. For the surface PD fault, the measured powers in each band were 0.1%, 99.1%, and 0.8%, and 89.2%, 10.7%, and 0.1%, in the lower, middle, and upper bands for the inner and outer antennas, respectively.

The cumulative energies for the bushing PD fault in the lower, middle, and upper bands were 69.3%, 27.4%, and 3.3% for the inner antenna, and 40.3%, 58.1%, and 1.6% for the outer antenna. For the two internal fault types, the majority of the cumulative power measured by the internal antenna was in the middle band, whilst for the external antenna, the majority of power was in the lower band. The inverse of this is the case for the bushing fault, where the majority of the power was in the middle band. The reason for this was due to the transformer tank causing low-pass filtering to the radiometric signal. However, the higher frequency content of the measurements made where the tank was not an obstruction was closely related. A measurement made at a distance greater than 3 m would likely have incurred attenuation to the higher frequency content.

In [38], the spectra of an emulated HV floating electrode PD fault, which is a common fault in switchgear [39], was obtained and compared using both galvanic and radiometric measurements.

A floating electrode PD test cell was energized to 6.2 kV using a HV DC supply. The measured energy was divided into bands of 50–290, 290–470, and 470–800 MHz. For the contact and radiometric measurements, the percentages of energy in each band were 62.8% and 78.3% in the 50–290 MHz band, 0.76% and 4.33% in the 290–470 MHz band, and 0.56% and 1.59% in the 470–800 MHz band, respectively, and the remaining energy was below 50 MHz. In both cases, the majority of the measured energy was within the 50–290 MHz band.

In [20,40], spectra were taken for a further two emulated PD faults using galvanic and radiometric measurements. Both PD cells emulated defects within solid insulation. One consisted of an acrylic tube containing two electrodes at each end, between the electrodes a sandwich of three circular pieces of 1.5 mm thick FR4 insulation are placed, in which the middle piece has a 1 mm hole drilled in the center to create a higher electric field within it, and therefore, a discharge. The second consisted of a sandwich of three sheets of 2.4 mm-thick epoxy glass dielectric between two electrodes, with a 1 mm diameter hole, again, drilled in the center of the middle sheet.

The acrylic tube PD source was filled with transformer oil in order to suppress any discharges occurring at the edges of the electrodes. However, measurements were taken with and without the presence of oil. The acrylic tube emulator was energized to 20 kV AC, both with and without oil, to generate PD, whilst the epoxy glass dielectric emulator was energized to 18 kV AC. Even though the frequency response of the acrylic tube emulator was higher with the presence of oil, almost the entire energy is in the 50–800 MHz band for all three measurements of the two emulators, with the majority of the energy being below 300 MHz.

### 2.2. Time Difference of Arrival PD Measurement

**Figure 5:**
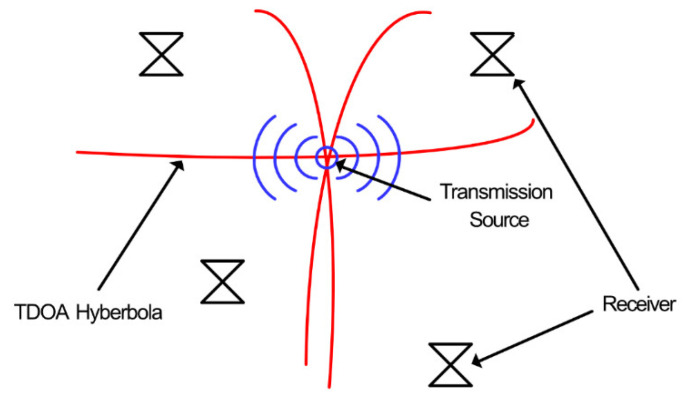
Time difference of arrival (TDOA) location of a wireless transmission with multilateration [42].

TOA and TDOA techniques, traditionally used for radio positioning systems, use the times that signals are received at specific receivers to determine the distance, using the speed of light to convert time to distance, of a transmitting signal from each receiver, and therefore, the location of the transmitting source [41]. TOA relies on the time at which the transmitted signal was sent along with the reception time for each receiver, whereas TDOA only requires the latter, and is therefore more versatile [42]. Figure 5 shows an example of TDOA positioning.

In this example, four receivers are positioned around a transmitting signal which is the target for location. The distance of the source from each receiver is calculated by determining the precise time difference of received signals between sensors. This is then converted to distance using the speed of light. The resulting distances are used to plot hyperbolic lines via non-linear regression, at which the intersecting point of the lines is the estimation for the transmission [43]. TDOA has been used in many schemes for PD location and can provide accuracy within 5 cm [44].

TDOA techniques have been employed for the detection of PD using multiple techniques, such as cross-correlation, cumulative energy, and the amplitude of the first received peak [45,46], that is, by comparing received PD patterns, or by using the integral of the received signal or the amplitude of the first received peak. TDOA location provides a high level of estimation accuracy and has been used to successfully detect and locate sources of PD in live HV environments [47,48,49,50,51].

Although TDOA techniques provide an accurate and non-invasive solution to PD detection and location, there are various constraints that impact the feasibility of large-scale deployment using this technology. The sample-rate required to discern the differences has to be as high as possible, since the time between samples equates to the resolution in distance [52]. Since the transmitted signal is a radiometric electromagnetic wave, the resolution in distance is proportional to the speed of light divided by the sample rate. For example, a sample rate of 1 GSa/s has a resolution distance of 30 cm, whereas a sample rate of 5 GSa/s has a distance resolution of 6 cm [53]. Whilst techniques such as interpolation can be used to increase the distance resolution at reduced sample rates [54,55], rates in excess of 1 GSa/s are still necessary.

The requirement for high sample rates makes this technology less attractive for a flexible large-scale battery powered wireless sensor network (WSN), since the power requirements of such a high-speed data processing system would not be capable of sustained operation from a single battery source over a reasonable period of time and would also have considerable costs. Furthermore, the complexity of scaling a coherent TDOA system on a large scale would be difficult, due to requirement for synchronization between nodes to accurately determine the time difference of signal reception.

### 2.3. Received Signal Strength PD Measurement

A simpler method to TOA and TDOA techniques is RSS only localization, which is based on the classical radiometric propagation model, given by Equation (1).

(1) $$R_{i}=R_{o}-10n\log_{10}d_{i}$$

where *R_i_* and *R_o_*, *n*, and *d_i_* are the *i*-th sensor received power and source transmitted power in dBm, path-loss index, and distance from the source, respectively. In free space, *n* is typically 2 at short distances from the transmitting source. It increases to approximately 4 when ground reflections occur, and it increases further still if the propagation environment contains obstructions or produces multi-path propagation.

The main benefit of an RSS-based system is the use of incoherent receivers, and therefore, no requirement for synchronization between nodes. This allows for a network that is far more flexible than coherent-based detection and can be easily scaled for a given monitoring area of HV plant [56]. This versatility comes at the cost of decreased accuracy compared to TOA and TDOA techniques, due to the complexity of the propagation environment [57]; however, this can be alleviated through calibration, by transmitting an artificial PD source of known strength from a single node within the WSN to each other node. The received signal at each node can then be adjusted to account for the propagation environment for a given transmission path [58]. A further challenge is the limited signal-to-noise ratio (SNR), due to the use of incoherent detectors with a wide bandwidth, and therefore a limited dynamic range.

A typical technique used in some proposed implementations [59,60,61] is to measure the energy of the received PD signal, since experimental data have been reported that link the total radio frequency (RF) energy propagated from a PD source to the apparent charge conducted across the void [62,63], suggesting that the two quantities have a linear relationship; therefore, the integral of the received PD power may contain useful diagnostic information.

Methods of RSS PD localization have been proposed which directly sample the received PD signal [64,65,66,67]. Whilst these techniques eliminate the need for synchronization, they still employ sample rates above 1 GSa/s. Although direct sampling of the received PD signal obtains detailed information of the PD signal, which allows for frequency analysis via fast-Fourier transform, along with pattern recognition and better noise immunity [66,68], it still requires excessive power consumption.

To reduce the required sample-rate, various techniques have been proposed which utilize envelope detection to remove the VHF/UHF frequency components, thus leaving only the envelope of the received PD signal [69], allowing for sample rates of approximately 20 MSa/s at a reduced measurement accuracy. Data published in [70] state an error of 0.54% at a sample rate of 100 MSa/s, with 20 samples per pulse for a 200 ns radiometric PD signal, compared to a sample-rate of 10 GSa/s and 20,000 samples per pulse. This accuracy drops to 8.68% at 10 MSa/s, with only two samples per pulse. Whilst this does not seem a significant error, due to the stochastic nature of the PD, it is likely that in some cases only one sample will be obtained for a received signal, therefore reducing the accuracy of the measurement.

**Figure 6:**
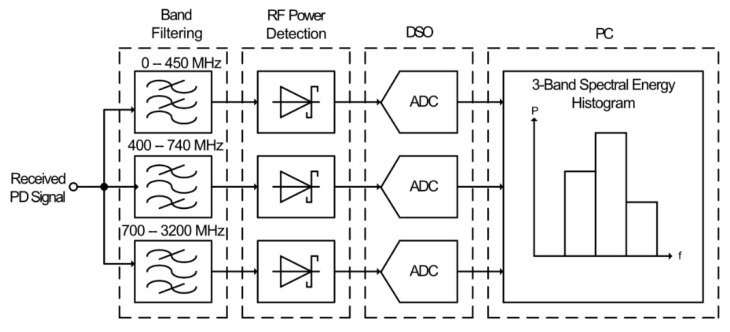
A frequency-based RF PD sensor [59].

A technique utilizing envelope detection, along with basic frequency measurement, was proposed in [59,71]. The proposed system, shown in Figure 6, used three parallel filtered channels to obtain basic spectra of the received PD signal.

The received RF signal is applied to three separate parallel filters, a 450 MHz cutoff low-pass filter, and 400–750 MHz and 700–3200 MHz band-pass filters. The filtered signals are each applied to a Schottky diode power detector with bandwidths of 5 MHz, which remove the UHF components from the received PD signal. The detected signals are then sampled at a rate of 1 GSa/s by a digital sampling oscilloscope (DSO). The sampled data are then processed via a PC to calculate the energy of received PD signals in each frequency band. This is then used to plot a histogram of the received pulses for a given energy band.

Whilst this system is only a prototype, it is limited for large-scale use due to various factors. The 1 GSa/s sample-rate would make the cost per sensor too high for a WSN to be a viable option for PD monitoring. Along with this, three ADCs are required to digitize each frequency band, increasing the cost and power consumption threefold. ADCs with a sample rate at least a factor of ten lower could be used to reduce this limitation. However, the data processing requirements would still require significant power consumption, particularly if the sampled data were transmitted back to the gateway before any processing was performed. Furthermore, the parallel connection of the RF front-end would result in the received signal power being split between each channel due to the 50 Ω matching, reducing the signal strength, and therefore the sensitivity of the sensor.

**Figure 7:**
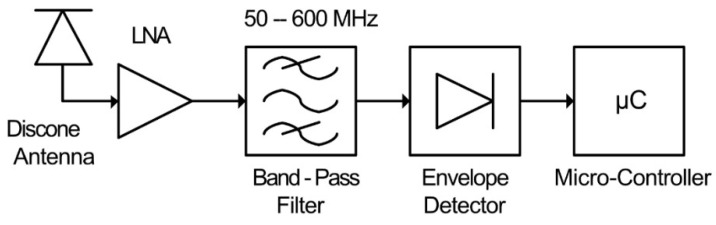
Single channel PD sensor proposed in [56].

A single channel sensor using envelope detection was proposed in [56], shown in Figure 7, which utilizes the internal ADC of a micro-controller to digitize the received PD.

The sensor is composed of a wideband receiving disk-cone antenna, LNA, band-pass filter, envelope detector and micro-controller. The disk-cone antenna is used to receive the radiometric PD signal. This is applied to an LNA to provide some amplification, increasing the sensitivity of the sensor. The amplified signal is band-limited to a bandwidth of 50–600 MHz via the band-pass filter, before being applied to the envelope detector, which removes the high frequency content from the PD signal. The envelope of the received PD is then sampled by the micro-controller’s internal ADC.

This arrangement alleviates the requirement for high-speed sampling, and thus, provides a low-power technique for radiometric PD monitoring. The envelope detector is a square responding device; therefore, each sample acquired by the micro-controller’s ADC is proportional to the power at a given point of the envelope of the received PD signal. The micro-controller can therefore process these samples to obtain the total received energy of the envelope. A disadvantage to this technique is the low sampling rate of the ADC, which decreases the accuracy of the measured PD signal, as well as there being no way to discern between received PD and other interfering signals.

**Figure 8:**
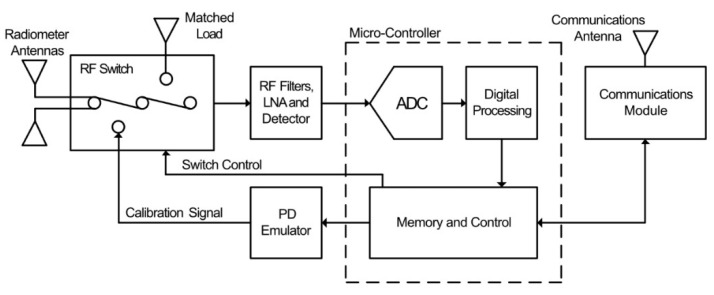
Advanced single channel PD sensor proposed in [72].

Further developments were made to the previous sensor, as proposed in [72]. The sensor, shown in Figure 8, is designed around the single channel version with the addition of calibration circuitry and a communications module for transmission of received data.

The receiving radiometer antenna is, again, a disk-cone type. Here, the radiometer antenna is connected to an RF switch, which configures the sensor as a receiver or transmitter. The signal processing section is nearly identical to that of Figure 7, with the exception that a logarithmic detector is used in place of the square-law detector, therefore providing higher sensor sensitivity and dynamic range. The main addition is the PD emulation circuitry, which is activated by the micro-controller in order to transmit a known PD-like signal to the other sensor nodes within the WSN. This allows for the propagation environment to be calibrated between nodes, increasing the accuracy of the radiometric localization. A further modification is the addition of a Zigbee communications module to transmit received data to a data collection point.

**Figure 9:**
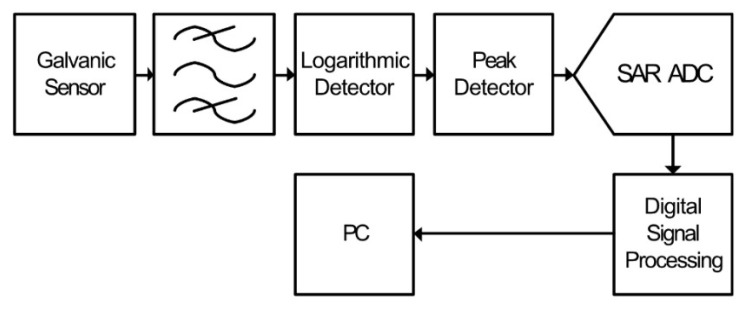
Peak detector-based PD signal processing.

A low power technique using an active peak-hold detector was proposed in [73]. This technique removes the requirement for multiple samples per pulse by holding the maximum peak of the received PD signal. Figure 9 shows the proposed system.

Whilst this technique is described for galvanic measurement of PD, it has potential for use in radiometric-based PD sensor. The sensor output is band-limited via the band-pass filter, the output of which is applied to a logarithmic detector. The logarithmic detector compresses the received signal, effectively increasing the dynamic measurement range. The output of the detector is then applied to an active peak detector, which captures and holds the maximum voltage of the detector output. This is then sampled by a 12-bit SAR ADC, which is internal to an ADuC814 data acquisition IC. Some digital signal processing is then applied to the digitized signal, and the result is transmitted to a PC via RS232.

The benefit of this technique is that only a single sample is required per received PD event, drastically reducing the required sample-rate, and therefore, power consumption and cost. A difficulty is that high-speed active peak hold circuits, capable of capturing peaks of less than 10 ns associated with radiometric PD signals, are difficult to implement. This is due in part to the requirement for rectifying diodes, which add non-linearity to the dynamic range of the detector. Further difficulties are caused by the requirement for high-speed FET input components, to ensure fast response and minimal hold droop, increasing the power consumption of the circuit. Furthermore, whilst the peak power of the received signal can be used for localization, it does not have a direct relationship to the apparent charge in the PD source [61]; therefore, diagnostic analysis using this technique is difficult.

### 2.4. Summary of the Reviewed Literature

This paper has outlined the techniques for the acquisition of short-duration analogue pulses, along with the advantages and limitations of each technique. The basic operation of relevant ADCs was discussed, with the suitability for analogue pulse acquisition, and in particular radiometric PD, being assessed. The type and functionality of typical RF envelope tracking detectors were described and evaluated. The typical frequency spectra for various radiometric PD faults were detailed, along with the frequency spectrum of locally transmitted signal that would likely be detected within the range of radiometric PD. Finally, techniques for radiometric partial discharge measurement were presented and discussed, with a focus on the suitability for their deployment in a large-scale PD monitoring WSN. The main points taken from this literature review are as follows:

- Whilst undersampling acquisition allows for a lower power ADC to be used, the lower sample rate limits the accuracy of the measurement. Conversely, oversampling provides a high level of measurement accuracy, but is limited by the requirement for higher power consumption.
- Schottky diode-based RF power detectors, e.g., LTC5507, can provide an accurate measurement of received power, at no or low power consumption, and can track the fast envelope of a received PD signal. However, their dynamic range is limited due to the requirement for them to operate in the square-law region of the diode.
- Logarithmic power detectors, e.g., AD8307, have high dynamic ranges, typically greater than 70 dB, and have sensitivities typically below −50 dBm. This comes at the cost of increased circuit complexity, and therefore, increased power consumption. Furthermore, they are limited by a response time in the range of hundreds of nanoseconds to microseconds.
- Various reported measurements have shown that the typical frequency spectra of many radiometric PD faults are within the 50–800 MHz band. Within this band there exist various transmitted signals that may cause interference to radiometric PD measurement.
- TOA and TDOA radiometric PD detection provides a high level of location accuracy, yet due to the requirement for high-speed sampling, in excess of 1 GSa/s and the requirement for synchronization between sensors, it is not suitable for large-scale monitoring of HV plants.
- RSS PD measurement techniques can alleviate the dependence on excessive conversion rates when used in conjunction with envelope detection. Furthermore, implementation of a large-scale WSN utilizing this technique is simpler because incoherent sensors can be used. However, existing sensors using RSS still require sample rates beyond 10 MSa/s.

## 3. Radiometric PD Detection Using TRI Based WSN

**Figure 10:**
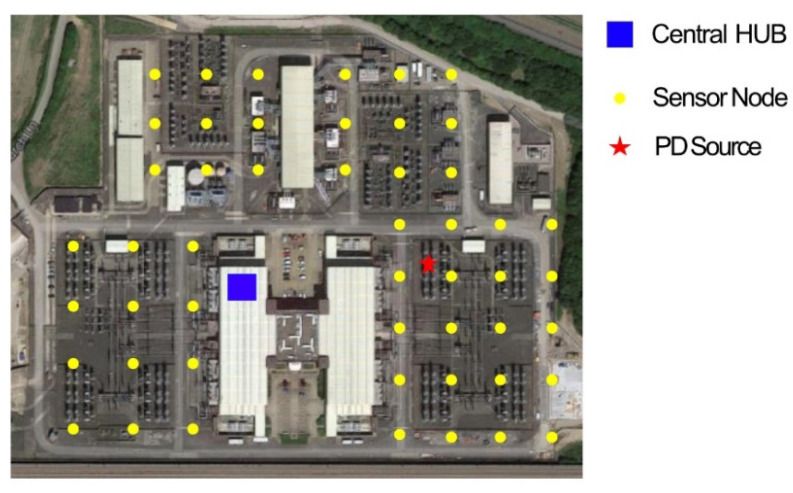
An example of a wireless sensor network (WSN) layout in a switchyard to detect PD using the prototype proposed in [74].

A prototype of radiometric PD detection system was proposed by Upton et al. in [74], which provides a solution to the disadvantages listed in the previous section. This prototype is composed of sensors nodes that communicate via a central Hub using the wirelessHART protocol. The sensor nodes are arranged in a grid array in a specified area of interest. WirelessHART may transmit data via intermediate nodes to the central HUB in order to ensure no data are lost by using the shortest transmission path available, and therefore, minimizing the possibility of a transmitted signal being beyond the receiver range of the central HUB due to attenuation over longer distances. The electromagnetically radiated PD signal is received by sensor nodes in the immediate vicinity of the source. The nodes measure the power of the PD. The average power measured at each sensor node is transmitted to the central HUB, where an RSS-based location algorithm [75,76] is used to estimate the location of the PD source, by triangulating the source based on the average power strengths received at each node. An example of this arrangement is shown in Figure 10.

**Figure 11:**
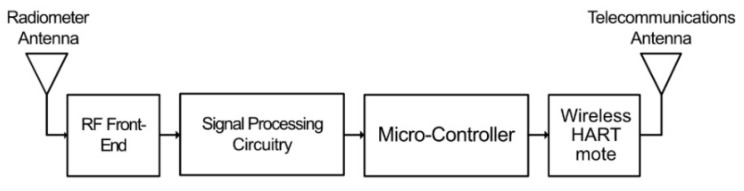
Block diagram of the sensor node of the PD detection system [74].

The sensor nodes of this system are developed to provide a system that is easy to install, low-cost, portable, and consumes as little power as possible. These nodes are designed to process and measure PD signals down to a level of at least −30 dBm, whilst also providing immunity to interference from broadcasting signals. A block diagram of the structure of the sensor node is shown in Figure 11.

**Figure 12:**
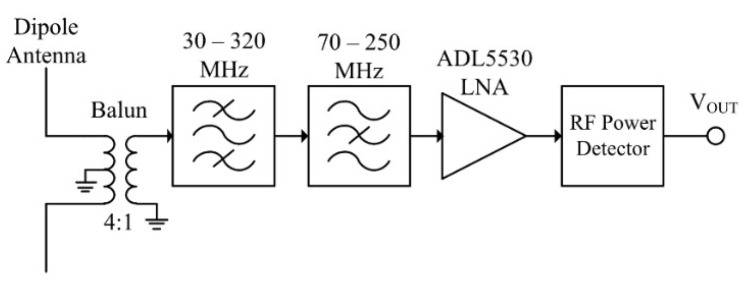
Block diagram of RF front-end.

The radiometric PD signal is received via a dipole antenna, connected via a 4:1 balun to provide a wider antenna bandwidth by providing a closer impedance match to the following section over a broader frequency range. A dipole antenna is used, as an omni-directional pattern is required. The received signal is then applied to the RF front-end block as shown in Figure 11. The RF front-end is represented as a block diagram in Figure 12.

**Figure 13:**
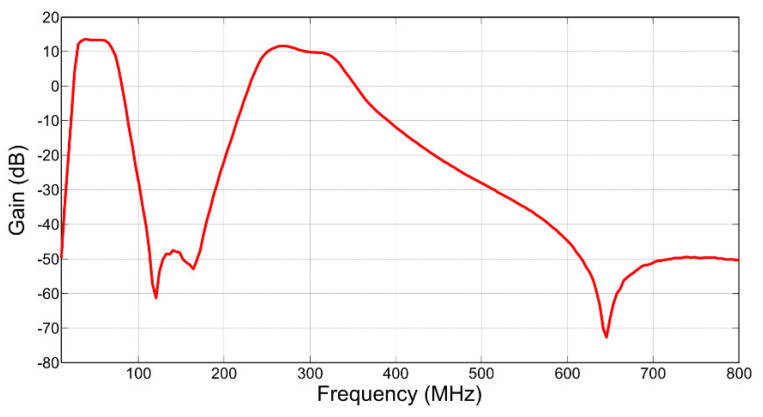
Frequency response of the RF front-end.

The signal is then applied to the filters and LNA. The band-pass filter removes interference and noise outside the 30–320 MHz band of measurement interest. Notably, it removes digital TV broadcasting signals and mobile communications signals in the UHF range. The band-stop filter removes FM, DAB, and other communications signals in the region of 70–250 MHz. The LNA then provides a gain of approximately 16 dB. The combined transfer function results in two passbands from 30 to 75 MHz and from 250 to 330 MHz, with a gain between 11.7 and 14.4 dB in the mid-band, and a measured noise figure between 5 and 7 dB [77]. The frequency response of the RF front-end is shown in Figure 13.

**Figure 14:**
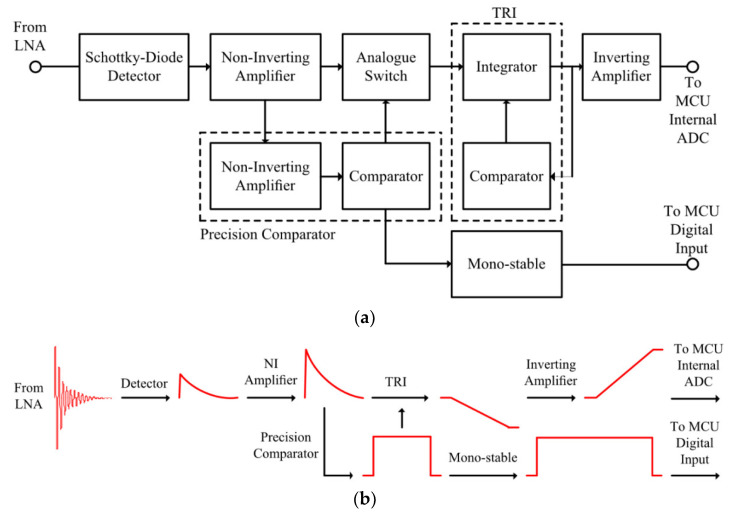
Transistor reset integrator (TRI): (**a**) block diagram and (**b**) signals [78].

The output signal from the LNA is then applied to an RF envelope detector, which removes the high frequency components and produces an output voltage proportional to the linear or logarithmic power of the received PD signal envelope, depending on the type of detector used. The envelope-detected PD signal is then applied to the signal processing section, which consists of an ADC [74] or of a composite transistor reset integrator (TRI), [78]. A block diagram and the signal representation of the TRI-based signal processing section is shown in Figure 14a,b, respectively.

**Figure 15:**
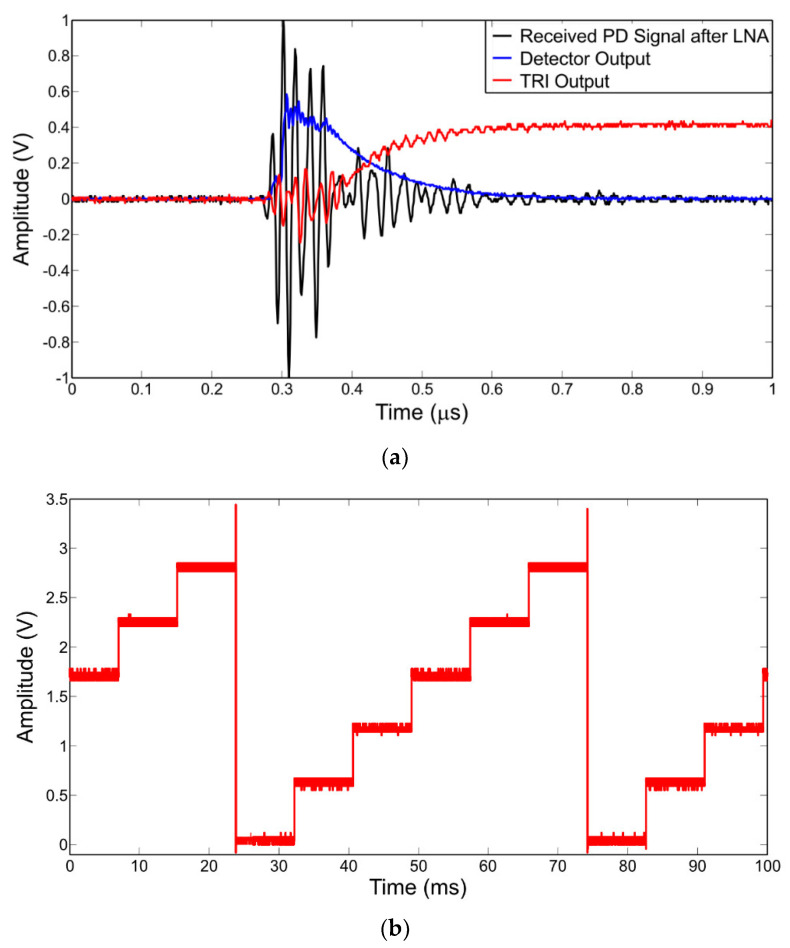
(**a**) TRI output for a single PD event and (**b**) TRI output for multiple PD events.

The output signal from the LNA is applied to an RF envelope square law detector, which removes the high-frequency components and produces an output voltage proportional to the instantaneous input power. The detected signal is then amplified by a high-speed NI amplifier to increase the amplitude of the signal to span the supply rail, which is then applied to the precision comparator and the TRI. The precision comparator is composed of a NI amplifier and a high-speed comparator. The NI amplifier increases the signal in order for the comparator threshold to be set to a voltage that is at least 40 mV above the noise floor, therefore ensuring that no oscillation occurs at the output of the comparator due to false triggering. When the output of the NI amplifier exceeds the threshold level, the comparator triggers high, activating the analog switch and producing a pulse at the output of the mono-stable circuit for a pulse count. The analog switch activates the TRI which integrates the received PD signal to a DC level. The TRI comprises a precision inverting integrator, a transistor, and a comparator. Each received PD signal causes a DC step change to the output of the integrator, proportional to the integral of the instantaneous power of the received pulse. Once the output voltage of the integrator reaches a predetermined level, the TRI comparator resets the TRI and the process is then repeated. The output of the TRI is then applied to an inverting amplifier to obtain a positive signal, which is then sampled by the internal successive approximation register (SAR) ADC of the micro-controller unit (MCU). In contrast to a standard TRI, a composite TRI technique involves utilization of composite multiple amplifiers that enhance performance beyond the capabilities of any single commercial amplifiers. Using composite TRI helps in improving speed and precision, and reduced power consumption compared to a standard TRI. A detailed circuit of the composite TRI signal processing section is presented in [78]. Figure 15a shows a received radiometric PD signal at the output of the LNA, the zero-bias detector output, and the TRI output, whilst Figure 15b shows the TRI output for multiple received emulated PD events.

The output of the signal processing section is then inputted into an MCU, which calculates the energy of the received radiometric PD signal and provides a count of the total number of PD events received over a predefined measurement period. The average of the received energy is then calculated by the MCU, and the mean received energy and count are transmitted back to the central HUB via a wireless HART transmitter. Since only two values are transmitted per measurement period, the requirement for data processing and memory are kept to a minimum, and therefore, power consumption is reduced. To ascertain the accuracy of the TRI-based signal processing systems for location estimation based on RSS, the data acquired by the system were then entered into a simple localization algorithm. The algorithm is based upon the ratio of received power for sensor node pairs [44], since the transmitted PD power is unknown. The algorithm uses the ratio of received sensor powers to estimate the distance of the PD source from the receiving nodes. The power ratio data from each sensor node are then processed to localize the PD source. Detailed calculations and explanations regarding the proposed localization algorithm are included in [75,76], along with comparisons with other existing algorithms. The proposed PD detection system and the localization algorithm were validated by performing two measurements: scenario 1: PD source localization using six sensors, and scenario 2: PD source localization using eight sensors. In both scenarios, the true location of the PD source from the origin is (13.5 m, 4.5 m), and is left unchanged.

**Figure 16:**
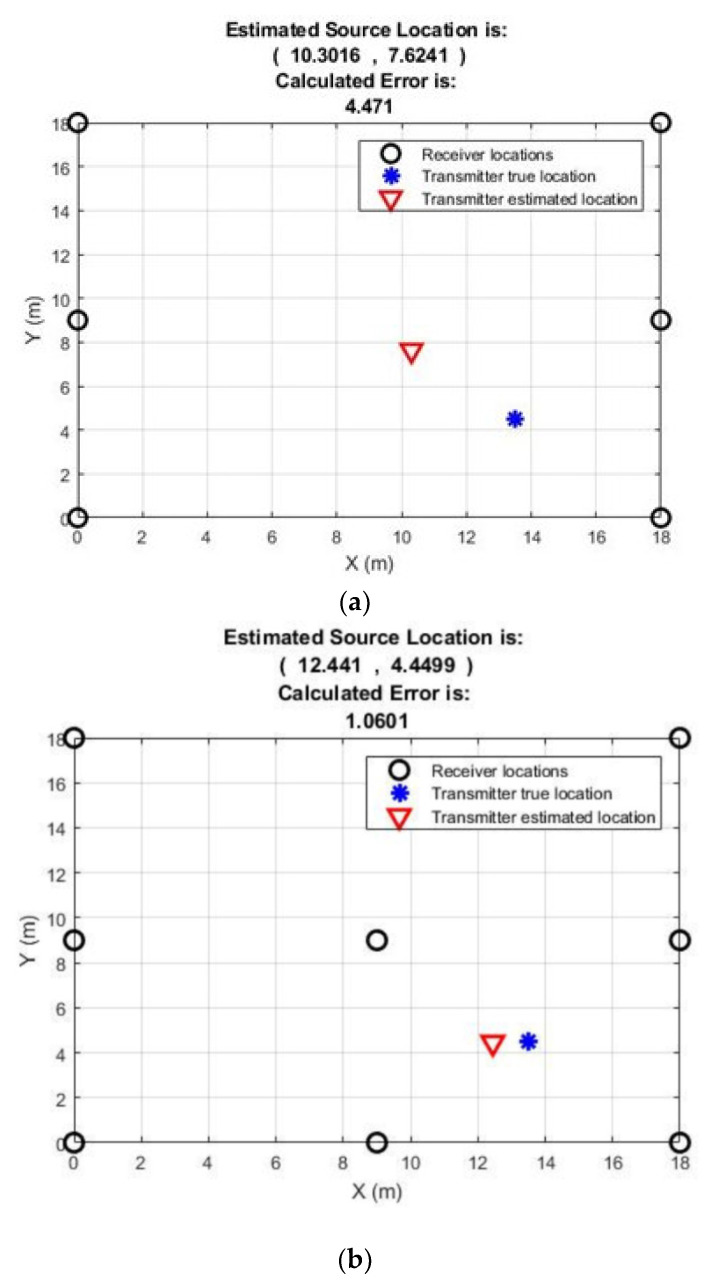
PD source detection using (**a**) six sensor nodes and (**b**) eight sensor nodes, [75].

The measurement setup of the PD detection system with the location of the receiving sensor nodes, the true location of the PD source, and the estimated location of the PD source using the localization algorithm is shown in Figure 16. The estimated coordinates of the PD source using six sensor nodes is (10.3 m, 7.6 m), and using eight sensor nodes it is (12.4 m, 4.4 m). The localization error using six sensor nodes is relatively high at 4.5 m, whereas the localization error using eight sensor nodes is only 1.06 m. This suggests that increasing the number of receiving sensor nodes used in the WSN greatly improves the localization accuracy of the system. Furthermore, the performance evaluation of the proposed algorithm and its comparison with other existing algorithms using several measurements is presented in [75]. It should be noted that most of the measurements for PD detection were carried out in outdoor environments; however, a few of the PD measurements were performed indoors. These indoor measurements might have radio reflections from the walls or other objects that generally reduce the accuracy of the localization algorithm.

## 4. Conclusions

This paper presents a detailed review of the techniques for the radiometric detection and localization of PD using received signal strength. Key advantages and disadvantages of the reviewed techniques are presented. Moreover, an overview of a radiometric technique using a TRI-based WSN is presented, which serves as a low-cost and power efficient solution to the drawbacks exhibited by radiometric PD detection techniques that rely on high-speed ADCs.

## Data Availability

Data can be made available with request to the correspondence.
